# Depletion of CABYR-a/b sensitizes lung cancer cells to TRAIL-induced apoptosis through YAP/p73-mediated DR5 upregulation

**DOI:** 10.18632/oncotarget.7069

**Published:** 2016-01-29

**Authors:** Qianqian Xiao, Zunlei Qian, Weiqing Zhang, Jin Liu, Enze Hu, Jinsan Zhang, Mingying Li, Junhao Wang, Fei Kong, Yunguang Li, Rui Wang, Xiaohua Tan, Dacheng He, Xueyuan Xiao

**Affiliations:** ^1^ Key Laboratory of Cell Proliferation and Regulation Biology, Ministry of Education, Beijing Normal University, Beijing, China; ^2^ College of Forensic Sciences, People's Public Security University of China, Beijing, China; ^3^ School of Pharmaceutical Sciences and Key Laboratory of Biotechnology and Pharmaceutical Engineering, Wenzhou Medical University, Wenzhou, Zhejiang, China; ^4^ Center of Reproduction and Genetics, First People's Hospital of Yunnan Province, Kunming, China

**Keywords:** CABYR-a/b, lung cancer cells, DR5, TRAIL, YAP

## Abstract

Our previous study revealed that knockdown of CABYR-a/b increases the chemosensitivity of lung cancer cells through inactivation of Akt. Here, we demonstrated that depletion of CABYR-a/b significantly increased DR5 expression and sensitized lung cancer cells to TRAIL-induced apoptosis *in vitro* and/or *in vivo*. Importantly, treatment with AD5-10, a DR5-specific agonistic monoclonal antibody, was able to mimic TRAIL-induced apoptosis in CABYR-a/b-silenced cells. Strikingly, we identified that depletion of CABYR-a/b not only increased the expressions of p73 and DR5 but also decreased the phosphorylation of YAP S127. Loss- or gain-of-function studies of YAP and p73 revealed that double deletions of YAP and p73 effectively decreased the expression of DR5 and abolished TRAIL-induced apoptosis in CABYR-a/b knockdown cells. Conversely, the co-overexpression of YAP and p73 promoted the expression of DR5 and sensitized cells to TRAIL-induced apoptosis. Taken together, our results demonstrate that depletion of CABYR-a/b sensitizes lung cancer cells to TRAIL-induced apoptosis through YAP/p73-mediated DR5 upregulation.

## INTRODUCTION

CABYR was first isolated from human spermatozoa and participates in the sperm capacitation. There are six transcript variants of CABYR, which encode five protein isoforms. CABYR-a and CABYR-b are the two predominant isoforms, and they have a high degree of nucleotide sequence similarity [1]. In our previous study, we verified CABYR as a novel cancer testis antigen in lung cancer [2]. In addition to lung cancer and brain tumors [2–3], CABYR was also shown to be aberrantly expressed in liver cancer and esophageal cancer [4]. Knockdown of CABYR-c in HepG2 cells resulted in the inhibition of cell growth [5]. However, few studies have evaluated the biological functions of CABYR in cancer cells, with the exception of studies performed in liver and lung cancer cells [4, 6]. Therefore, the full biological functions of CABYR in cancer cells have yet to be elucidated.

The tumor necrosis factor-related apoptosis-inducing ligand (TRAIL) is a powerful inducer of apoptosis in a wide range of human cancer cell lines [7–10]. To date, five receptors have been identified for TRAIL, and only DR4 and DR5 have cytoplasmic death domains that engage extrinsic or intrinsic apoptotic pathways upon TRAIL binding [11]. Although, intrinsic resistance mechanisms to TRAIL-induced apoptosis are not fully understood, numerous mechanisms have been described, including alteration of death receptors or Akt activation [12–13]. Therefore, molecules or agents that can modulate these mechanisms of TRAIL resistance could improve the therapeutic efficacy of TRAIL.

Our previous study established that depletion of CABYR-a/b inhibited the proliferation and increased the chemosensitivity of lung cancer cells *in vitro* and *in vivo* via inhibition of the Akt pathway [6]. Strikingly, it has been reported that Akt phosphorylates the Yes-associated protein (YAP) at serine 127. YAP S127 interacts with the 14–3–3 protein in the cytoplasm and results in the attenuation of p73-mediated cell apoptosis [14–15]. Moreover, growing evidence in the literature describes YAP as a transcriptional co-regulator of p73-mediated apoptosis in cancer cells [16–18]. The present study provides the first evidence that depletion of CABYR-a/b decreases YAP S127 phosphorylation and upregulates the expression of p73 and DR5, thus increasing TRAIL-induced apoptosis in lung cancer cells.

## RESULTS

### Depletion of CABYR-a/b sensitizes lung cancer cells to TRAIL-induced apoptosis

**Figure 1:**
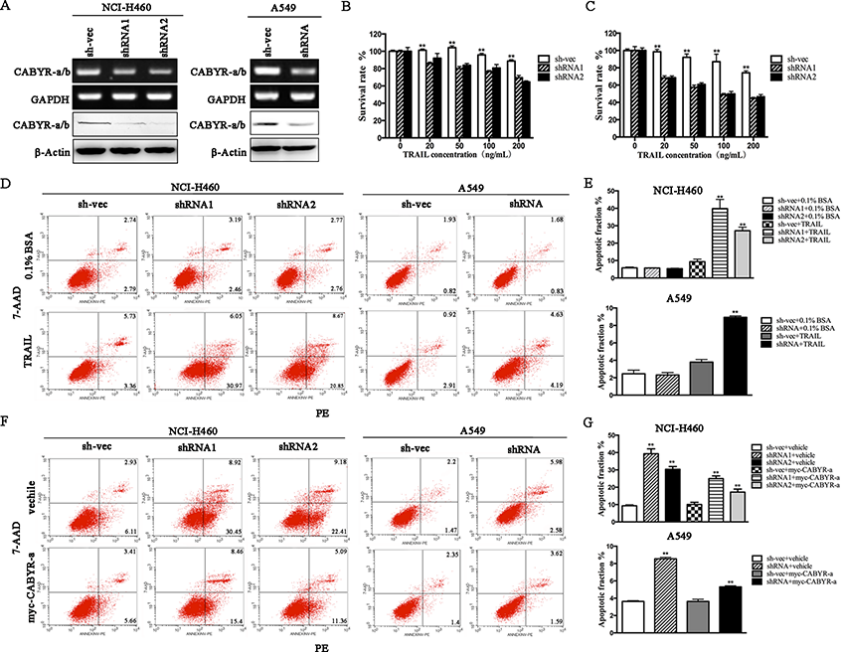
Silencing of CABYR-a/b enhances TRAIL-induced apoptosis in lung cancer cells Expression of CABYR-a/b in H460 and A549 cells was determined by RT-PCR and western blotting (**A**). Cell survival rates were measured by the MTT assay following treatment with the indicated concentrations of TRAIL for 12 h (**B**) or 24 h (**C**) in CABYR-a/b-silenced and control H460 cells. The cell survival percentage was obtained in comparison with that of the respective control cells. The apoptotic percentage was measured using PE/7-AAD staining and analyzed by flow cytometry in CABYR-a/b-silenced and control cells treated with 20 ng/mL TRAIL for 6 h in H460 cells or 200 ng/mL for 24 h in A549 cells. 0.1% BSA was used as negative control (**D**). The histograms demonstrate the apoptotic rates of NCI-H460 and A549 cells (**E**). Re-introduction of CABYR-a in shRNA cells partially restored cells TRAIL resistance in response to similar concentrations of TRAIL (**F**). Histograms demonstrate the apoptotic rates of cells (**G**). The results represent the means of at least three independent experiments. *p < 0.05 and **p < 0.01 for the comparisons of the experimental and control groups. β-Actin was used as an internal control to ensure equal protein loading.

To explore the effect of CABYR on TRAIL-induced apoptosis in lung cancer cells, two predominant isoforms, CABYR-a and CABYR-b, were simultaneously silenced by siRNA due to their high degree of nucleotide sequence similarity (referred to as ‘CABYR-a/b’). Stable knockdown CABYR-a/b was established in H460 and A549 lung cancer cell lines (Figure 1A), which express higher levels of endogenous CABYR-a/b compared to other lung cancer cell lines tested. Stable CABYR-a/b-silenced H460 cell clones (shRNA1 and shRNA2) and a control cell clone (sh-vec) were treated with different concentrations of TRAIL for 12 or 24 h, and cell survival rate was assessed using the MTT method. Interestingly, depletion of CABYR-a/b alone did not induce cell apoptosis (data not shown) but significantly potentiated the cytotoxic effects of TRAIL in a dose- and time-dependent manner (Figure 1B–1C). A similar phenomenon was also observed in CABYR-a/b-silenced A549 cell clone (shRNA) (data not shown). Subsequently, we confirmed that the decrease of aforementioned TRAIL-induced survival in CABYR-a/b-depleted cells was a result of increased apoptosis as evidenced by Annexin V-PE/7-AAD staining. The TRAIL-induced apoptotic rate was increased by more than six-fold in shRNA1 cells and five-fold in shRNA2 cells compared to sh-vec cells (Figure 1D–1E). Importantly, knockdown of CABYR-a/b in A549 cells, which have been reported to be resistant to TRAIL treatment [19], increased the TRAIL-induced apoptotic rate more than two-fold compared with sh-vec cells (Figure 1D–1E). Representative flow cytometry results were shown in Figure 1D. Subsequently, to confirm that CABYR-a/b was required for TRAIL-induced apoptosis in lung cancer cells, we selected CABYR-a and performed a rescue experiment in CABYR-silenced cells and the corresponding control cells. Notably, ectopic expression of CABYR-a significantly reversed TRAIL-sensitivity in CABYR-a/b-depleted cells (Figure 1F–1G). Collectively, our results strongly suggest a negative correlation between the expression level of CABYR-a/b and TRAIL-induced apoptosis in lung cancer cells.

### Silencing of CABYR-a/b increases *in vivo* tumor sensitivity to TRAIL

**Figure 2:**
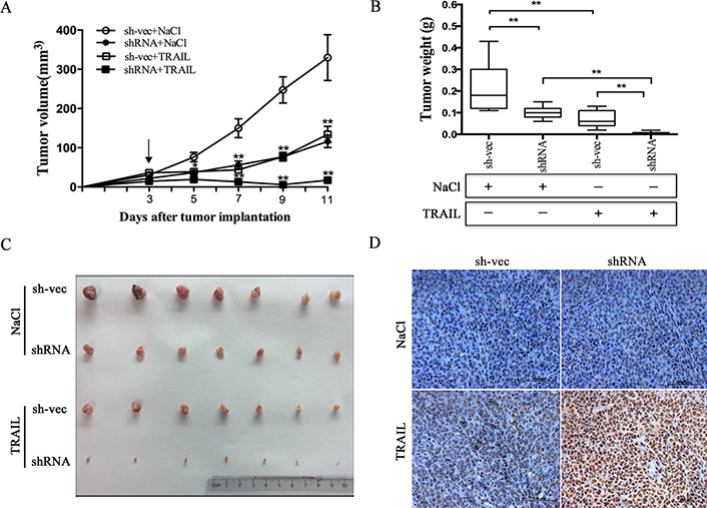
Suppression of CABYR-a/b increases *in vivo* tumor sensitivity to TRAIL The tumor xenograft volumes were measured on the indicated day, and the mice were treated with 0.9% NaCl or TRAIL for 11 days after inoculation; the arrow (“↓”) indicates the day of drug administration (**A**). Tumors were weighed after the mice were euthanized, and the mean ± SD of the tumor weights are presented (**B** and **C**). **P < 0.01 for the comparison of the CABYR-a/b-shRNA combination and the CABYR-a/b-shRNA alone groups. Apoptosis was evaluated by TUNEL assay (**D**). Scale bar = 20 μm.

We next verified whether silencing of CABYR-a/b can sensitize cells to TRAIL-mediated apoptosis *in vivo*. H460 shRNA1 and sh-vec cells were implanted into the left and right flank areas of nude mice, and TRAIL (10 mg/kg) was given via the tail vein after tumor diameters reached 0.3 cm. The drug was administered once every other day. Consistent with our *in vitro* results, the mean tumor volume in the animals rejected with CABYR-a/b-silenced cells and treated with TRAIL was significantly lower as compared to tumors observed in the corresponding single-treatment groups (*p < 0.05) after 5 days (Figure 2A). Moreover, these animals showed the lowest tumor weight among all of the groups (Figure 2B–2C). Next, we used TUNEL analysis to confirm that the observed reduction in tumor size was due to increased apoptosis in shRNA1 and sh-vec plus TRAIL treatment groups. However, the proportion of apoptotic cells was significantly higher in the CABYR-a/b-silenced plus TRAIL treatment group compared to the single TRAIL treatment in sh-vec cells (Figure 2D). Similar to the results obtained *in vitro*, single depletion of CABYR-a/b had only a minor effect on inducing cell apoptosis *in vivo*. These results indicate that the downregulation of CABYR-a/b also sensitizes lung cancer cells to TRAIL *in vivo* through the induction of apoptosis.

### Knockdown of CABYR-a/b increases TRAIL-induced apoptosis by upregulation of DR5

**Figure 3:**
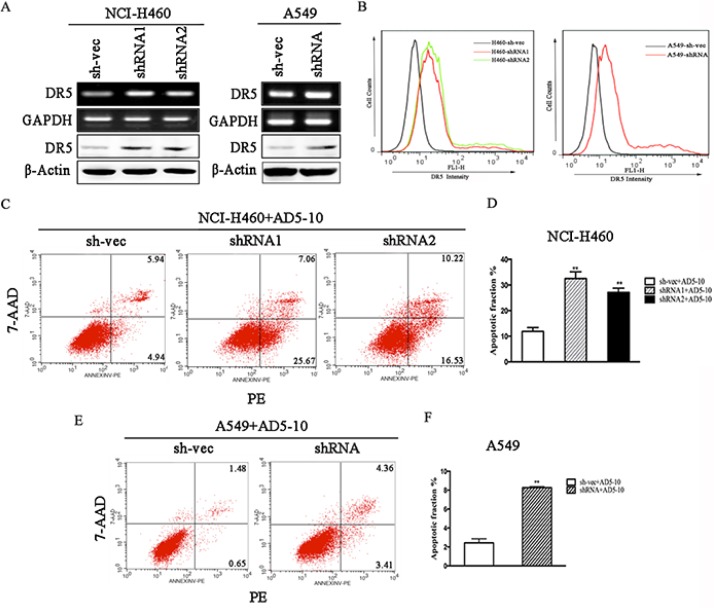
Knockdown of CABYR-a/b induces the expression of DR5 Expression of DR5 was determined by RT-PCR and western blotting in CABYR-a/b-silenced H460 and A549 cells (**A**). Cell surface expression of DR5 was measured by immunofluorescent staining with a DR5-specific antibody and was analyzed by flow cytometry (**B**). The apoptotic percentage was measured using PE/7-AAD staining and analyzed by flow cytometry in CABYR-a/b-silenced and control cells treated with 200 ng/mL AD5-10 for 24 h in H460 cells and A549 cells, respectively (**C, E**). Histograms demonstrate the apoptotic rates within these cells. The results represent the means of at least three independent experiments (**D, F**). The experimental cell survival percentage was obtained in comparison with that of the respective control cells. **P < 0.01 was obtained for the comparison of the CABYR-a/b-shRNA and sh-vec groups. β-Actin was used as an internal control to ensure equal protein loading.

To explore the underlying mechanism through which CABYR-a/b inhibits TRAIL-induced apoptosis in lung cancer cells, we examined the expression of death receptors DR4 and DR5. As expected, depletion of CABYR-a/b increased the expression of DR5 at both the mRNA and protein levels in cells (Figure 3A), whereas no significant induction of other TRAIL receptors was observed (data not shown). An increase in cell surface expression of DR5 was also observed in CABYR-a/b -silenced cells using a flow cytometry assay with a specific anti-DR5 antibody (Figure 3B). The control IgG antibody did not display similar results (data not shown). To further validate that DR5 upregulation underlies TRAIL-induced apoptosis in CABYR-a/b-silenced cells, we treated CABYR-a/b-silenced and control cells with an agonistic DR5 monoclonal antibody, AD5-10, which has been reported to specifically bind to DR5 and induce cancer cell apoptosis [20]. Importantly, AD5-10 treatment also dramatically enhanced apoptosis in CABYR-a/b-silenced H460 cells compared to sh-vec cells (Figure 3C–3D). Strikingly, the average rate of apoptosis in A549 cells was shown to increase by three-fold in shRNA cells compared to sh-vec cells (Figure 3E–3F). The nonspecific IgG did not cause cell apoptosis (data not shown). Thus, the upregulation of DR5 is a crucial event in sensitizing CABYR-a/b-depleted cells to TRAIL-induced apoptosis.

### Deletion of CABYR-a/b increases p73 expression and decreases YAP S127

**Figure 4:**
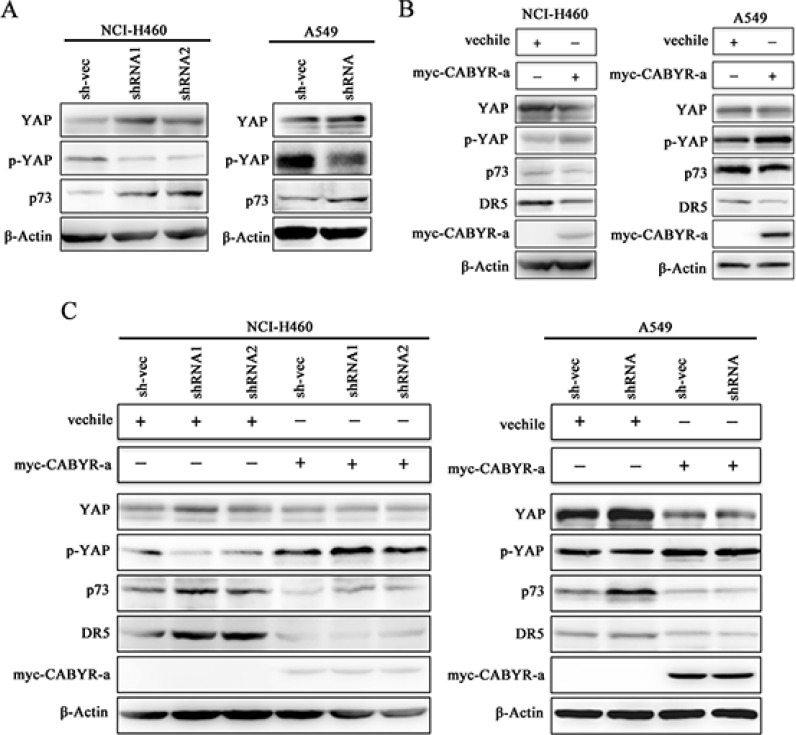
Depletion of CABYR-a/b promotes YAP phosphorylation and p73 expression The expression of YAP and p73 in the silenced CABYR-a/b and control cells was detected by western blotting in H460 and A549 cells (**A**). Wild type H460 and A549 cells (**B**), as well as cells stable depleted for CABYR-a/b (**C**) were transiently transfected with a plasmid vehicle or plasmid encoding myc-CABYR-a. YAP, p73 and DR5 were subsequently analyzed by western blotting. An antibody against myc was used to detect the overexpression of CABYR-a. β-Actin was used as an internal control to ensure equal protein loading.

YAP acts as a coactivator of p73, and phosphorylation of YAP at serine 127 (Ser127) impairs YAP-nuclear translocation and attenuates p73-mediated pro-apoptotic gene expression [16]. To examine how CABYR-a/b inhibits DR5 expression in lung cancer cells, we examined the expression of YAP and p73. As shown in Figure 4A, silencing of CABYR-a/b resulted in decreased YAP S127 levels and an upregulation of p73 expression. Conversely, overexpression of CABYR-a in wild-type cells resulted in an increase in YAP S127 phosphorylation and decreased the expression of p73 and DR5 (Figure 4B). Interestingly, re-introduction of CABYR-a via transient transfection in CABYR-a/b-silenced cells partially restored TRAIL resistance and YAP phosphorylation but decreased the expression of p73 and DR5 compared to control vehicle-transfected cells (Figure 4C). Together, these results imply that CABYR-a/b may repress the expression of DR5 through YAP/p73.

### Depletion of CABYR-a/b up-regulates DR5 expression and sensitizes cells to TRAIL-induced apoptosis through YAP/p73 cooperation

**Figure 5:**
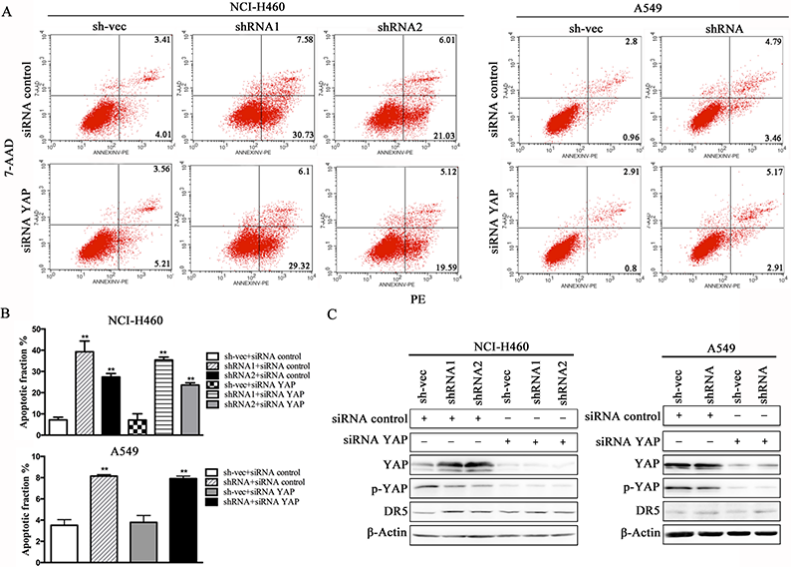
Suppression of YAP does not decrease TRAIL-induced apoptosis in cells that are stably silenced for CABYR-a/b Knockdown of YAP using siRNA for 48 h in H460 and A549 cells that were stable silenced CABYR-a/b. Scramble siRNA was used as a control. These cells were treated with 20 ng/mL TRAIL for 6 h in H460 cells or 200 ng/mL for 24 h in A549 cells. The percentage of apoptosis was measured using PE/7-AAD staining and then was analyzed by flow cytometry (**A**). Histograms demonstrate the apoptotic rates of the cells (**B**). The results represent the means of at least three independent experiments. The expression of YAP, p73 and DR5 was detected by western blotting (**C**). β-Actin was used as an internal control to ensure equal protein loading.

**Figure 6:**
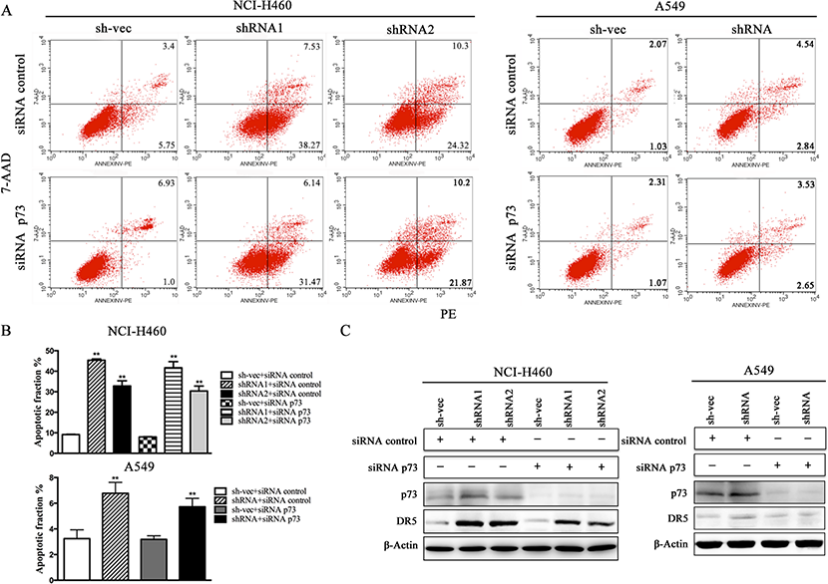
Silencing of p73 does not inhibit TRAIL-induced apoptosis in cells that are stably silenced for CABYR-a/b Deletion of p73 using siRNA for 48 h in H460 and A549 cells that were stably silenced CABYR-a/b. Scramble siRNA was used as a control. These cells were treated with 20 ng/mL TRAIL for 6 h in H460 cells or 200 ng/mL for 24 h in A549 cells. The percentage of apoptosis was measured using PE/7-AAD staining and was analyzed by flow cytometry (**A**). Histograms demonstrate the apoptotic rates of the cells (**B**). The results represent the means of at least three independent experiments. The expression of YAP, p73 and DR5 was detected by western blotting (**C**). β-Actin was used as an internal control to ensure equal protein loading.

**Figure 7:**
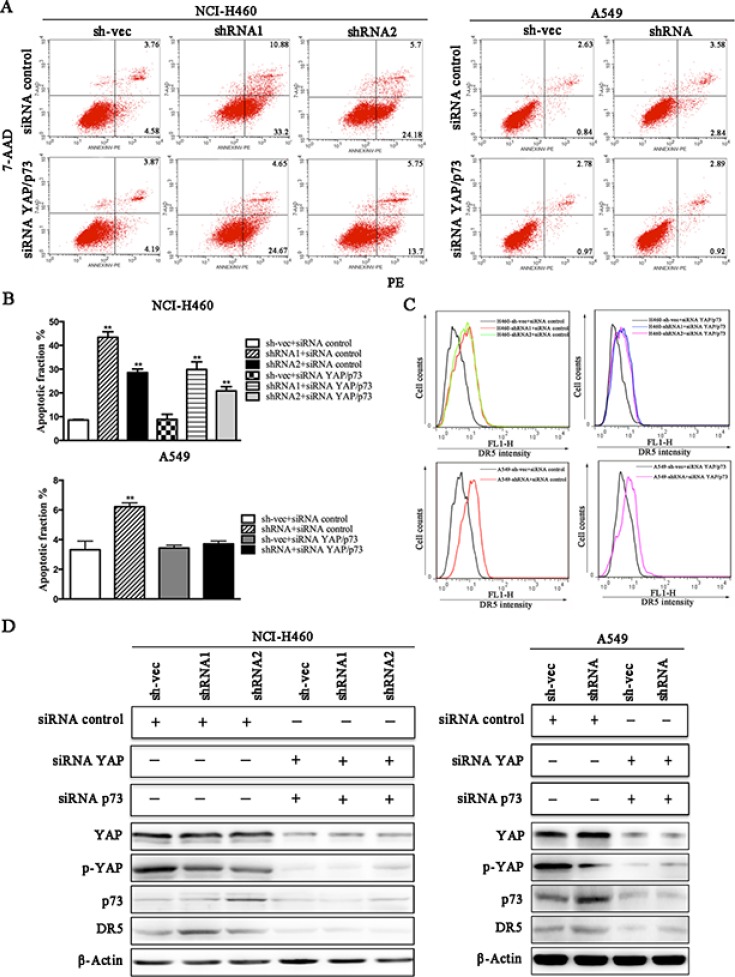
Knockdown of CABYR-a/b up-regulates the expression of DR5 and sensitizes cells to TRAIL-induced apoptosis through YAP/P73 cooperation CABYRa/b-silenced and control cells were treated with 20 ng/mL TRAIL for 6 h in H460 cells or 200 ng/mL for 24 h in A549 cells after transfection with the combination of siRNA targeting YAP and p73. Scramble siRNA was used as a control. The percentage of apoptosis was measured using PE/7-AAD staining and was analyzed by flow cytometry (**A**). Histograms demonstrate the apoptotic rates of cells transfected with both siRNA YAP and siRNA p73 (**B**). The results represent the means of at least three independent experiments. The expression of YAP, p73 and DR5 was detected by western blotting (**C**). β-Actin was used as an internal control to ensure equal protein loading.

**Figure 8:**
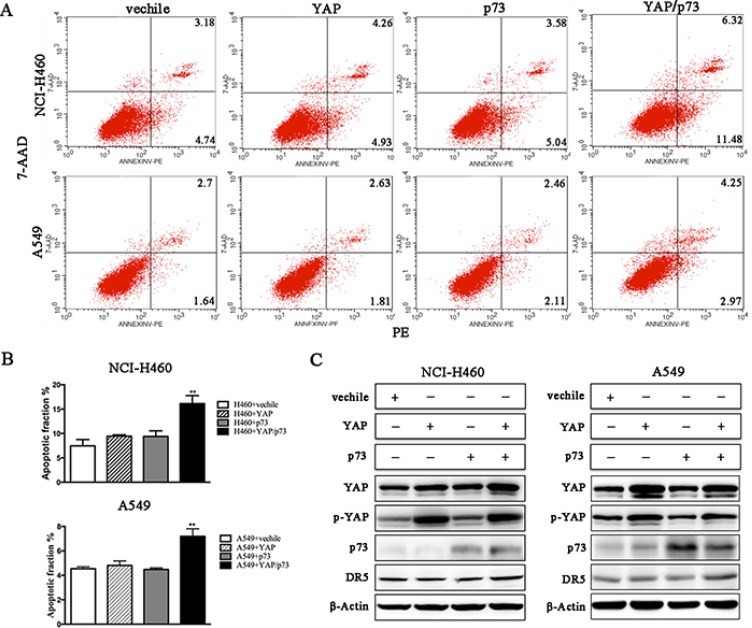
Co-overexpression of YAP and P73 promotes TRAIL-induced apoptosis Wild-type H460 and A549 cells were treated with 20 ng/mL TRAIL for 6 h or 200 ng/mL for 24 h, respectively, after transient transfection with the indicated plasmids. The percentage of apoptosis was measured using PE/7-AAD staining and was analyzed by flow cytometry (**A**). The histograms demonstrate the apoptotic rates of the cells, and the results represent the means of at least three independent experiments (**B**). The expression of YAP, p73 and DR5 was detected by western blotting (**C**). β-Actin was used as an internal control to ensure equal protein loading.

We conducted a loss-of-function study to further confirm the role of YAP/p73 in TRAIL-induced apoptosis. CABYR-a/b-silenced and control cells were transfected with specific siRNAs targeting YAP, p73 or both. Scramble siRNA was used as a control. Efficient knockdown of YAP and p73 was validated by western blotting in Figures 5C and 6C. Interestingly, single removal of endogenous YAP (Figure 5A–5B) or p73 (Figure 6A–6B) in CABYR-a/b-depleted cells had only a minor effect on TRAIL-induced apoptosis. However, the combined abrogation of endogenous YAP and p73 expression in these cells led to a severe reduction in DR5 expression and inhibited TRAIL-induced apoptosis (Figure 7A–7D). Conversely, co-overexpression of YAP and p73, but not single overexpression of YAP or p73, upregulated DR5 expression and sensitized cells to TRAIL-induced apoptosis in wild-type cells (Figure 8A–8C). These results strongly support our hypothesis that DR5 is regulated by CABYR-a/b through YAP/p73 cooperation.

## DISCUSSION

TRAIL is a member of the TNF superfamily and preferentially induces apoptosis in transformed or malignant cells without affecting normal cells. By binding to death receptors, such as DR5, TRAIL induces apoptosis in a wide range of human cancer cell lines via activation of extrinsic or intrinsic apoptotic pathways [21]. In the present study, we found that silencing of CABYR-a/b sensitized lung cancer cells to TRAIL-induced apoptosis both *in vitro* and/or *in vivo*. Based on the above-mentioned reports and our results, we hypothesize that death receptors may be involved in CABYR-a/b-mediated resistance to TRAIL treatment. To substantiate this possibility, we first examined the TRAIL-associated death receptors in CABYR-a/b-silenced and control cells. As expected, depletion of CABYR-a/b up-regulated the expression of DR5 at both the mRNA and protein levels and increased the cell surface expression of DR5 in cells. However, the expression of DR4 and two other decoy receptors was not affected (data not shown). If DR5 is required for CABYR-a/b -mediated resistance to TRAIL treatment, activation of DR5 should mimic TRAIL to induce apoptosis in these cells. Subsequently, CABYR-a/b-silenced and control cells were treated with an agonistic DR5 monoclonal antibody (AD5-10), which has been reported to specifically bind to DR5 and induce cancer cell apoptosis [20]. As expected, treatment with AD5-10 also enhanced cell apoptosis. Thus, our findings support the notion that upregulation of DR5 is critical to sensitize CABYR-a/b-silenced cells to TRAIL-induced apoptosis.

p73 is known to regulate the transcription of pro-apoptotic proteins, such as DR5 [22]. Recently, a close link between YAP and p73 has emerged. Some reports show that YAP engages in a physical association with p73 and is a selective co-activator of p73-mediated transcription of pro-apoptotic genes [23]. To date, YAP-mediated promotion of p73 activity has only been investigated in the context of DNA-damage signaling [24]. To investigate whether CABYR-a/b represses the expression of DR5 through the p73/YAP axis, we examined the expression of p73 and YAP in CABYR-a/b -silenced and control cells. Strikingly, depletion of CABYR-a/b significantly promoted p73 expression and suppressed YAP S127 in shRNA cells. If the expression of DR5 is upregulated by p73/YAP cooperation in shRNA cells, loss- and gain-of-function of YAP and p73 in shRNA cells should decrease or increase DR5 expression and TRAIL-induced apoptosis. Indeed, simultaneous depletion of YAP and p73, but not the suppression of either protein alone, dramatically inhibited DR5 expression and TRAIL-induced apoptosis in CABYR-a/b knockdown cells. In contrast, co-overexpression of YAP and p73 increased TRAIL-induced apoptosis and also increased the expression of DR5 in wild-type lung cancer cells. However, the expression of YAP or p73 was insufficient to sensitize TRAIL-induced cell apoptosis. Collectively, we demonstrate that depletion of CABYR-a/b increases DR5 expression via the YAP and p73 transcriptional axis.

With the exception of the Hippo pathway, YAP is phosphorylated at serine 127 by Akt [14–15]. Because our previous study showed that silencing of CABYR-a/b resulted in Akt inactivation, we wondered whether Akt might also activate YAP phosphorylation and inhibit TRAIL-induced apoptosis in CABYR-a/b-silenced cells. Unexpectedly, although transfection with Myr-Akt completely restored YAP phosphorylation in CABYR-a/b -silenced cells, it did not suppress the expression of DR5 and p73 or TRAIL-induced apoptosis (data not shown). This finding suggests that other potential alternative pathways may be regulated by CABYR-a/b in response to p73 expression, which is beyond the scope of the present study.

Taken together, we demonstrate for the first time that depletion of CABYR-a/b up-regulates DR5 expression and sensitizes TRAIL-induced apoptosis through the YAP/p73 transcriptional axis in lung cancer cells. Our findings provide new insight into the functions of CT antigens in cancers. We could use siRNA specifically targeting CABYR-a/b as a small molecule drug to treat cancers. Alternatively, the combination of TRAIL with chemotherapy promotes cancer cell apoptosis while also decreasing the toxicity of drugs.

## MATERIALS AND METHODS

### Cells culture

NCI-H460 and A549 cell lines were purchased from the Academy of Sciences Committee Type Culture Collection Cell Bank and were authenticated by short tandem repeat analysis at the HK Gene Science Technology Co (Beijing, China). Cells were cultured in DMEM containing 10% FCS, penicillin (100 units/mL), streptomycin (100 units/mL), and 2 mmol/L glutamine and grown at 37°C in a humidified atmosphere with 5% CO_2_.

### Reagents, antibodies and plasmids

Recombinant Human TRAIL/Apo2 ligand (rhTRAIL) was purchased from Peprotech (Rocky Hill, USA). The Annexin V-PE/7-AAD Kit was obtained from BD Pharmingen (USA). Polyvinylidene difluoride membranes were obtained from Millipore (Bedford, MA, USA). Lipofectamine^™^ 2000 and jetPRIEM Transfection Reagent were purchased from Invitrogen (USA) and Polyplus (France), respectively. The antibody against Death Receptor 5 (DR5) was purchased from Abcam (Hong Kong, Ltd.). An agonistic DR5 monoclonal antibody (AD5-10), which has been reported to specifically bind to DR5 and then induce cancer cell apoptosis, was kindly provided by Dr. Zheng (Institute of Basic Medical Sciences (IBMS) of Chinese Academy of Medical Sciences, China) [23]. The antibodies against β-actin, p73, and YAP were purchased from Santa Cruz Biotechnology (Santa Cruz, CA, USA). The antibody against p-YAP S127 was purchased from Cell Signal Technology. Mouse anti-CABYR-a/b antisera were prepared in our laboratory. Secondary antibodies, goat horseradish peroxidase (HRP)-conjugated anti-rabbit IgG and anti-mouse IgG were purchased from Dakocytomation (DAKO Ltd., Ely, United Kingdom). 3-(4, 5-Dimethyl-2-thiazolyl)-2, 5-diphenyl-2-H-terazolium bromide (MTT) was obtained from Merck (Darmstadt, Germany). pGPH1/Neo-CABYR-a/b plasmids and a control shRNA scrambled vector, as well as YAP and p73 small interfering RNAs were purchased from Gene Pharma. pcDNA4-His-YAP plasmids were kindly provided by Dr. Zhang (Mayo Clinic College of Medicine, USA). The other plasmids used in the present study were preserved in our laboratory.

### Cell transfection

For the generation of stably transfected cells, pGPH1/Neo-CABYR-a/b-121 and pGPH1/Neo-CABYR-a/b -303, which effectively silenced CABYR-a/b gene expression, were transfected into NCI-H460 and A549 cells using Lipofectamine^™^ 2000 Transfection Reagent (USA/Invitrogen) according to the manufacturer's protocol. Scrambled vector was used as the control. After 24 h, the cells from each transfection were split into three separate culture dishes to ensure that independent lines were established. Stable cell lines were selected by growth in the presence of 0.4 mg/mL G418 (Merck Darmstadt, Germany), and individual cell lines were isolated using cloning discs (PGC Scientific, Frederick, MD). To knockdown the expression of YAP and p73, specific small interfering RNAs were used, and the targeted sequences were as follows: CCAGACGUGAUGACAAGAUTT (YAP-1662) and AUCUUGUCAUCACGUCUGGTT (p73-615). Scrambled siRNA was used as the control. To ensure the consistency of transfection in each experiment, H460 and A549 cells were counted and plated in 24-well and 6-well plates before transient transfection. The JetPRIME Transfection Reagent (Polyplus) was used according to the manufacturer's protocol.

### RT-PCR

Total RNA was extracted from cells for the generation of single-stranded cDNA and PCR was performed using specific primers to detect the expression of CABYR-a/b and Death Receptor 5. The primers used for each gene were as follows: CABYR-a/b (forward: 5′-CGGAATTCATTTCTTCAAAGCCCAGACTT-3′, reverse: 5′-TCATGGGCCCTTATTCAGCTGTTGATTC), Death Receptor 5 (forward: 5′-TAAAGGTGGCTAAA GCTGAGGCAGC-3′, reverse: 5′-TCAAAGTACGCACA AACGGAATGAT-3′) and glyceraldehyde-3-phosphate dehydrogenase (forward: 5′-AGGTCGGAGTCAACGGA TTTG-3′, reverse: 5′-GTGATGGCATGGACTGTGGT-3′).

### Western immunoblot

Western blot analysis was performed as described in our previous study [6]. Briefly, cells were lysed and equal amounts of sample proteins were separated by 10% SDS-PAGE, followed by transfer to a polyvinylidene difluoride membrane. The membranes were incubated with the primary antibodies overnight at 4°C. The primary antibodies were used at dilutions of 1:100 to 1:1000. Secondary antibodies conjugated to HRP were added and detected with a chemiluminescent substrate. β-Actin was used as an internal control for equal protein loading.

### MTT assay

Cell viability was analyzed by the MTT assay. Cells were seeded in 96-well plates at 8 × 10^3^ cells in 100 μL of complete medium per well. After overnight incubation, various concentrations of rhTRAIL (0–200 ng/mL) were added to each well, and 0.1% BSA was added as a control. The subsequent steps were performed as described in our previous study [6]. Each condition was tested in four replicates.

### Flow cytometry

Apoptosis was measured by flow cytometry. Staining was carried out by suspending 1 × 10^5^ cells in 0.5 ml of Annexin V-PE/7-AAD according to the manufacturer's protocol. To examine cell surface TRAIL receptor expression, a primary antibody to DR5 was used, followed by a secondary FITC-conjugated antibody. Mouse IgG antibody was used as an isotype control. Flow cytometry was conducted with a FACScan instrument (BD Biosciences). The data presented are representative of at least three different experiments.

### Xenografts and drug treatment

All animals used in this study were female BALB/C nude mice (4–6 weeks old) purchased from Charles River Laboratories (Wilmington, MA) and raised at the Peking University Laboratory Animal Center (Beijing, PR China). Each mouse was injected with stably silenced CABYR-a/b cells and mock control cells (2 × 10^6^/100 μL) into the left and right flank areas, respectively. When the tumor diameter reached 0.3 cm, the mice were randomly divided into three groups (seven mice per group): (i) 0.9% NaCl (control group) and (ii) 0.9% NaCl plus rhTRAIL (10 mg/kg). The drug or 0.9% NaCl was administered into the tail vein once every other day. Tumor growth was also monitored once every other day and was calculated as described in our previous study [6]. All mice were killed 2 days after the last treatment, and the total number and total weight of the tumors from each mouse were examined. All animal work was approved by the Ethics Committee of the College of Life Science, Beijing Normal University (CLS-EAW-2014-011). All of the methods were conducted in accordance with the relevant guidelines by the above Ethics Committee. Statistical analysis was performed using the Mann-Whitney two-sided test and Student's *t*-test.

### TUNEL assay

Apoptosis was detected in tumor tissue sections using the terminal deoxynucleotidyl transferase-mediated dUTP nick-end labeling (TUNEL) enzyme reagent (Fisher Scientific, Pittsburgh, PA, USA) according to the manufacturer's instructions. Digital images were captured by microscopy using an upright Zeiss Axio Imager.

### Statistical analysis

The experimental results are expressed as the mean ± SD. All experiments were repeated in duplicate or more. The experimental groups and their corresponding control groups were analyzed with two-tailed Student's *t*-test using GraphPad software. Differences with a *P* value of < 0.05 were considered statistically significant.
